# The Diagnostic Value of High-Frequency Ultrasound in Unclarified Lesions at the End of Extremities

**DOI:** 10.3390/diagnostics15131605

**Published:** 2025-06-25

**Authors:** Haojie Yang, Na Ni, Weiwei Ren, Qiao Wang, Mengyu Lu, Yincheng Gao, Guanqun Zhang, Yongxian Lai, Yujing Zhao, Lehang Guo, Dandan Shan, Liping Sun

**Affiliations:** 1 Department of Medical Ultrasound, Shanghai Tenth People’s Hospital, School of Medicine, Tongji University, Shanghai 200040, China; 15290548513@163.com (H.Y.); rww9456@163.com (W.R.); wangqiao078@163.com (Q.W.); lumengyu168@163.com (M.L.); gaoych22@163.com (Y.G.); zhanggq801@163.com (G.Z.); gopp1314@hotmail.com (L.G.); 2 Shanghai Engineering Research Center of Ultrasound Diagnosis and Treatment, School of Medicine, Tongji University, Shanghai 200040, China; 3 Department of Medical Ultrasound, Shanghai Skin Disease Hospital, School of Medicine, Tongji University, Shanghai 200443, China; 4 Department of Surgery, Shanghai Skin Disease Hospital, School of Medicine, Tongji University, Shanghai 200443, China; nnkkfuji@163.com (N.N.); laiyx@shskin.com (Y.L.); 5 Department of Medical Imaging, Shanghai Skin Disease Hospital, School of Medicine, Tongji University, Shanghai 200443, China; zhaoyujing6551@163.com

**Keywords:** ultrasound, dermatology, extremity, lesions, high-frequency ultrasound

## Abstract

**Objectives**: Unlike other body parts, unclarified lesions at the end of extremities have unique challenges due to their small size and interference. Traditional imaging methods struggle with low resolution. HFUS enhances resolution, offering a potential diagnostic value. **Methods**: From January 2019 to October 2023, the clinical and HFUS data of patients with unclarified lesions at the end of extremities were retrospectively analyzed. Independently, the diagnosis was made using two diagnostic modes (Mode A: only clinical information; Mode B: clinical and HFUS information). The diagnostic performance of the two modes was evaluated across different classification methods. **Results**: For all lesions, the correct rate of Mode B was higher than that of Mode A (52.8% vs. 18.4%, *p* < 0.001), and the indeterminate rate decreased by 43.0%. For benign lesions (51.0% vs. 18.2%), subungual lesions (40.8% vs. 21.1%), non-subungual lesions (55.6% vs. 17.8%), and common cases (60.9% vs. 20.3%), the diagnostic correct rate of Mode B was also higher than that of Mode A (all *p* < 0.05). However, there was no significant difference in rare lesions (9.8% vs. 4.9%) and malignant lesions (62.9% vs. 19.4%) between the two modes (both *p* > 0.05). Moreover, the indeterminate rate for all categories of lesions significantly diminished. Otherwise, Mode B demonstrated strong performance for malignant lesions (85.7% vs. 42.9%, *p* < 0.001). **Conclusions**: Adding HFUS can significantly improve the accuracy of diagnosing unclarified lesions at the end of extremities and reduce uncertainty, especially for benign and common lesions. HFUS has also demonstrated better performance in screening for malignant lesions.

## 1. Introduction

The lesions at the end of the extremities (fingers and toes) not only affect the patient’s daily work but also can lead to amputation and death [1,2,3,4]. However, current clinical studies mainly focus on lesions in the head, face, trunk, perineum, and proximal parts of the limbs while ignoring lesions at the end of the extremities [5,6,7,8,9]. It should be pointed out that the disease spectrum at the end of the extremities is different from the other parts mentioned above. In addition, it should be pointed out that current clinical research on lesions in the extremities is more focused on single-disease studies and analysis of a certain type of subungual lesion. Therefore, there are few comprehensive studies on the disease spectrum of lesions in the extremities [10,11,12]. The relevant experience based on other parts cannot be well applied, resulting in difficulties in diagnosis. The lesions at the end of extremities are complex and diverse; there are differences in the treatment of benign and malignant lesions [13]. Therefore, finding an accurate management method to manage lesions at the end of the extremities is necessary.

Pathological biopsy remains the gold standard for diagnosing soft tissue lesions at the end of extremities, albeit accompanied by drawbacks of invasiveness and a time-intensive process. The use of noninvasive diagnostic methods has recently increased, including dermatoscopy, CT, MRI, and ultrasound. Dermatoscopy has limitations in evaluating the intricate internal structural details of the lesions [14]. Both CT and MRI offer valuable insights into the internal architecture of lesions, but their relatively low resolution poses a significant challenge for precise evaluation, particularly given the typical attributes of soft tissue lesions in the limbs, especially their small volume and superficial location. The inherent limitations of traditional imaging methods make accurate diagnosis of such lesions a persistent challenge [15,16,17,18].

Due to the limitations of the above examination methods, it is difficult to diagnose the lesions accurately. Therefore, most lesions at the end of the extremities rely only on clinicians’ physical examination. High-frequency ultrasound (HFUS) is known for its safety, cost-effectiveness, superior soft tissue resolution, and ability to visualize blood flow signals. Its affordability ensures broader patient access, and its high resolution enables early detection and characterization of lesions. With the continuous increase in ultrasound frequency, HFUS, as a potent clinical tool with the potential to enhance diagnostic accuracy, can facilitate early intervention and, ultimately, improve patient outcomes [19,20,21]. However, there is no study on HFUS for lesions at the end of the extremities. Thus, we conducted this study to explore the diagnostic performance of HFUS for lesions at the end of the extremities.

## 2. Method

The institutional ethics committee (Shanghai Dermatology Hospital Clinical Trial Ethics Committee, 25 May 2023) has approved this study (SSDH-IEC-SG-029-4.1) and does not require informed consent from patients.

### 2.1. Patients

We retrospectively collected 438 patients at the Shanghai Dermatology Hospital from January 2019 to October 2023. These patients sought medical attention due to the lesions at the end of the extremities.

**Figure 1:**
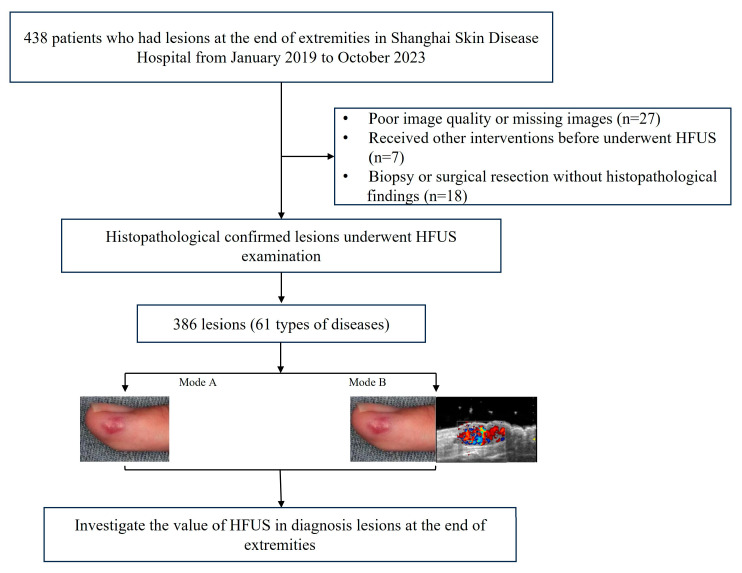
The patient selection flowchart. These images are from a male patient with an angioma at the end of his finger. Mode A: only clinical information was used; Mode B: physical examination information and HFUS information were used.

Based on the inclusion and exclusion criteria, we identified 386 lesions that fulfilled the specified research requirements (Figure 1).

The inclusion criteria were as follows:

(I) Having grayscale images, color Doppler flow imaging (CDFI), and dynamic scanning images simultaneously;
(II) Histopathological results from biopsy or surgical resection as the gold standard.

The exclusion criteria:

(I) Poor image quality;
(II) The histopathological results were unclear;
(III) Patients received other interventions before undergoing HFUS examination.

### 2.2. Acquisition and Analysis of HFUS Findings

All patients underwent examination with HFUS transducers. A total of 5–10 frames of grayscale images and CDFI images were collected for each patient. Dynamic video footage was also captured. These HFUS transducers were included: 6–20 MHz (Supersonic Imagine Aixplorer, Aix-en-Provence, France), 10–22 MHz (My Lab TMC class C, Genova, Italy), and 22–38 MHz (Kolo, Suzhou, China). The specific inspection method is as follows.

(I) Before each examination, the radiologist helped the patients position their bodies in a way that fully exposed the lesions.
(II) If the lesion was located superficially, the radiologist applied sufficient amounts of gel and gently placed the transducer on the lesion’s surface.
(III) Appropriate pressure was applied to the transducer for deep or large lesions to ensure high-quality lesion imaging. Each lesion was thoroughly scanned during the examination, and adjustments were made to the gain, depth, and focus to display each lesion.
(IV) CDFI parameters were also adjusted to suppress noise artifacts and display color Doppler flow signals.

### 2.3. Clinical Diagnoses

All HFUS images were de-identified (coded by patient ID only). Then, two radiologists with equivalent expertise (>5 years) independently diagnosed all lesions using two distinct modes (Mode A and Mode B).

(I) Mode A: only referenced clinical information, which includes features such as clinical manifestations, regional palpation, past medical history, and treatment history for diagnosis.
(II) Mode B: the combination of clinical information and HFUS image information, which includes assessing the lesion’s size, boundary clarity, internal echo characteristics, the characteristics of the skin layer, the involved range of the lesion, and CDFI features.

They were unaware of each other’s diagnostic results and the pathological results of the lesion before diagnosis.

Pathological examination results served as the gold standard to ensure objectivity and precision. Based on pathological findings, the diagnostic outcomes of the two modes were classified into correct and incorrect diagnoses, while indeterminate diagnoses encompassed scenarios where diagnoses were either absent or ambiguous, reflecting a lack of definitive diagnostic clarity.

### 2.4. Classification Analysis

The occurrence of diseases in various locations, with varying probabilities, is intricately intertwined with their specific pathological characteristics. Furthermore, physicians’ different degrees of familiarity with distinct diseases contribute to the complexity of diagnosis. We employed varied categorization schemes to gain a nuanced understanding of the unique prevalence of lesions in the extremities and assess the diagnostic accuracy among different radiologists. Specifically, the common diseases predominate, necessitating a nuanced understanding of their prevalence in clinical practice, so lesions were classified into common (*n* ≥ 5) and rare (*n* < 5) diseases, enabling a distinct analysis of the diagnostic efficacy between these prevalent and rarer lesion types.

Mode B can through nails to observe the internal structure of the lesions, the surrounding soft tissue conditions, and blood flow richness. Therefore, Mode B shows a certain potential in diagnosing complex lesions. We classified the lesions into subungual and non-subungual lesions. Additionally, given the potential for variability in disease manifestations between lesions on fingers and toes, we conducted a separate analysis for each anatomical site, thereby acknowledging the potential for differing disease types and ensuring a nuanced approach to understanding and managing these conditions in the future.

The differentiation between benign and malignant lesions is paramount in clinical decision-making as it necessitates distinct management strategies tailored to the nature of the lesion. Accurately distinguishing between the two categories is crucial for ensuring appropriate treatment, prognosis assessment, and patient outcomes. Therefore, based on pathological examination, the lesions were divided into benign and malignant lesions. Additionally, it should be pointed out that there were some lesions that proved invisible to HFUS, but they were diagnosed as indeterminate lesions according to clinical information.

### 2.5. Statistical Analysis

Statistical analysis was performed using IBM SPSS software (version 25.0, IBM Corporation, Armonk, NY, USA). The chi-square and McNeale tests analyze the differences between the two diagnostic modes. (When researching a single disease, we classify uncertain diagnoses as incorrect and use the McNeale test method for analysis because of the limited number of lesions.) *p* < 0.05 was considered statistically significant. In addition, in terms of calculating diagnostic performance, receiver operating characteristic (ROC) curves were used to calculate the area under the ROC curve (AUC) values for the two diagnostic modes, and the differences between the AUC values were identified using the Delong test method.

## 3. Results

### 3.1. Baseline Characteristics

A total of 438 patients with soft tissue lesions at the end of extremities from the Shanghai Dermatology Hospital received HFUS examination between January 2019 and October 2023. Based on the inclusion and exclusion criteria, 386 patients (mean age 50.4 ± 20.1 years, range 3 to 95) were included. There were 154 males and 232 females, and each patient had only one lesion included in the study (Table 1). A total of 61 kinds of diseases were diagnosed, including malignant melanoma (8.3%, 32/386), cutaneous squamous cell carcinoma (5.2%, 20/386), digital mucous cyst (14.2%, 55/386), thecal cyst (4.1%, 16/386), angioneoplasm (13.4%, 52/386), giant cell tumor of tendon sheath (2.6%, 10/386), and other rare diseases (*n* < 5) (Table 2).

### 3.2. Performance of Different Diagnosis Modes

There were 77 lesions that were diagnosed as indeterminate in Mode B. However, it should be pointed out that due to the resolution of HFUS, 51 lesions with extreme thicknesses were invisible in HFUS and were recognized as indeterminate lesions (51/77, 66.2%). For the other visible lesions (*n* = 335), the diagnostic performance of Mode B was better than that of Mode A (Table 3, *p* < 0.001). For Mode A, 19.4% of lesions were diagnosed correctly (65/335), 17.3% of lesions were misdiagnosed (58/335), and 63.3% of lesions were diagnosed as indeterminate (212/335). For Mode B, the correct diagnosis was improved to 60.9% (204/335). The misdiagnosis rate was 31.3% (105/335). Furthermore, the indeterminate rate was reduced to 7.8% (26/335).

**Figure 2:**
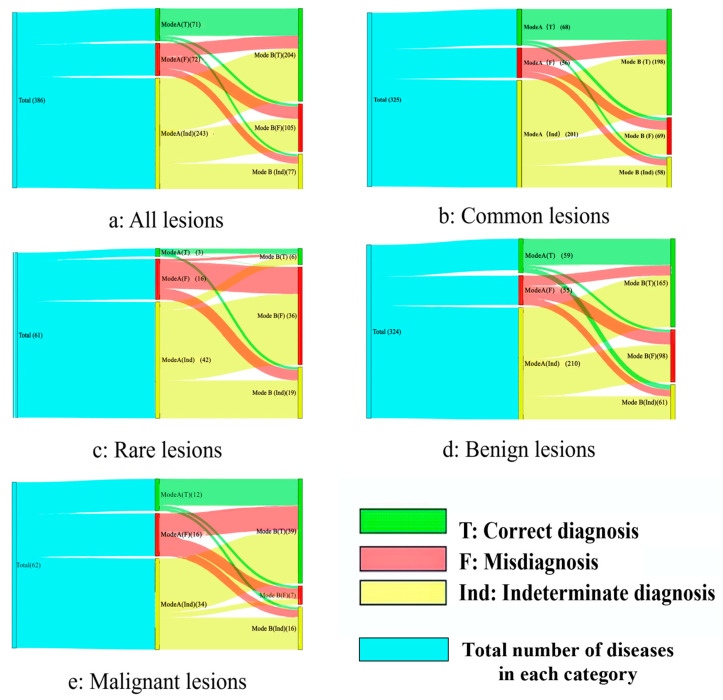
The Sankey diagram for Mode A and Mode B. (**a**) All 386 lesions; (**b**) common cases; (**c**) rare cases; (**d**) benign lesions; (**e**) malignant lesions. The Sankey diagram shows the distribution and flow direction of the lesion after diagnosis using Mode A and Mode B. Taking Figure (a) as an example, the blue strip represents the gold standard diagnostic result. A total of 71 lesions were diagnosed correctly using Mode A, while 60 lesions were diagnosed correctly, 6 lesions were diagnosed incorrectly, and 5 lesions were diagnosed as uncertain using Mode B. Please refer to Figure A1 in Appendix A, for specific distribution information.

In addition, when we analyzed all 386 lesions, Model B still showed satisfactory performance (*p* < 0.001) (Table 2, Figure 2a). For Mode A, 18.4% of lesions were diagnosed correctly (71/386), 18.7% of lesions were misdiagnosed (72/386), and 62.9% of lesions were diagnosed as indeterminate (243/386). For Mode B, the correct diagnosis rate was 52.8% (204/386). The misdiagnosis rate was 27.2% (105/386), and the indeterminate rate was 20.0% (77/386).

### 3.3. Common and Rare Lesions

There were 325 common lesions (325/386, 84.2%). Using Mode A, there were 68 lesions (68/325, 20.9%) that were correctly diagnosed, 56 lesions (56/325, 17.2%) that were misdiagnosed, and 201 lesions (201/325, 61.9%) that were diagnosed indeterminately. Using Mode B, 198 lesions (198/325, 60.9%) were correctly diagnosed, 69 lesions (69/325, 21.2%) were misdiagnosed, and 58 lesions (58/325, 17.9%) were diagnosed indeterminately. The correct rate of Mode B is higher than that of Mode A (*p* < 0.001). In rare diseases, though Mode B performed better than Mode A (9.8% [6/61] vs. 4.9% [3/61]), the two modes had no discernible difference in diagnostic efficacy (*p* = 0.06). (Table 3, Figure 2b,c).

**Figure 3:**
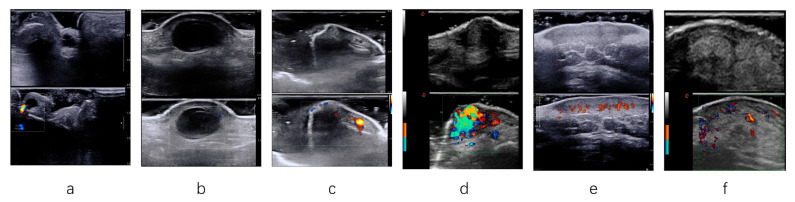
Figures (**a**–**f**) show the ultrasound image features of digital mucous cyst, thecal cyst, wart, angioneoplasm, malignant melanoma, and cutaneous squamous cell carcinoma in sequence. (**a**) B-mode sonogram showing an echoless mass with regular margins. CDFI sonogram showing no vascularity in the lesion. (**b**) B-mode sonogram showing an echoless mass with regular margins and adjacent to the thecal cyst. CDFI sonogram showing no vascularity in the lesion. (**c**) B-mode sonogram showing a hypo-echoic flat mass with irregular margins; there is sound attenuation behind the lesion. CDFI sonogram showing no vascularity in the lesion. (**d**) B-mode sonogram showing a hypo-echoic mass. CDFI sonogram showing extremely rich increased vascularity in the lesion. Transient blood flow signal enhancement is visible in the compressed lesion. (**e**) B-mode sonogram showing a hypo-echoic mass. The lesion grows downward from its base, exhibiting an invasive behavior. CDFI sonogram showing increased vascularity in the tissue. (**f**) B-mode sonogram showing an iso-echoic mass with irregular and blurred margins. Excessive keratinization of the lesion surface. CDFI sonogram showing increased vascularity in the mass.

In addition, when analyzing specific diseases, Mode B also showed a higher diagnosis performance in diseases such as digital mucous cyst, cutaneous squamous cell carcinoma, malignant melanoma, wart, thecal cyst, and angioneoplasm (all *p* < 0.05) (Table 2, Figure 3).

### 3.4. Benign and Malignant Lesions

There were 5 kinds of malignant diseases in all 61 types of diseases (malignant melanoma, cutaneous squamous cell carcinoma, Bowen’s disease, malignant proliferative dermal papilloma, and porokeratosis, *n* = 62, 62/386, 16.1%). There were 12 lesions (12/62, 19.4%) and 39 lesions (39/62, 62.9%) correctly diagnosed by Mode A and B, respectively. Mode B’s correct rate was higher than Mode A’s. However, the difference was not statistically significant (*p* = 0.198). The remaining 324 lesions (324/386, 83.9%) were benign lesions. A total of 59 lesions (59/324, 18.2%) and 165 lesions (165/324, 51.0%) were correctly diagnosed through Mode A and B, respectively. The correct rate of Mode B was higher than that of Mode A (*p* < 0.001). It should be noted that in terms of benign and malignant diagnosis, the indeterminate rate of Mode A was greater than 50% (54.8% [34/62] and 64.8% [210/324]), respectively, while Mode B reduced the diagnosis indeterminate rate (25.8% [16/62] and 18.8% [61/324]) (Table 3, Figure 2d,e).

### 3.5. Subungual and Non-Subungual Lesions

There were 71 lesions (71/386, 18.4%) of subungual lesions. There were 15 lesions (15/71, 21.1%) that were correctly diagnosed using Mode A, and 29 lesions (29/71, 40.8%) were correctly diagnosed using Mode B. For the other 315 lesions (315/386, 81.6%) of non-subungual lesions, there were 56 lesions (56/315, 17.8%) that were correctly diagnosed by Mode A and 175 lesions (175/315, 55.6%) that were correctly diagnosed using Mode B. For both subungual and non-subungual lesions, the diagnostic performance of Mode B was higher than that of Mode A (*p* = 0.009 and *p* < 0.001) (Table 3).

### 3.6. Anatomical Site Analysis

There were 247 lesions (247/386, 63.9%) that occurred on the finger. There were 55 lesions (55/247, 22.3%) and 140 (140/247, 56.7%) lesions that were diagnosed correctly by Mode A and B, respectively (*p* < 0.001). For the other 139 lesions (139/386, 36.1%) of the toe, there were 16 lesions (16/139, 11.5%) and 64 lesions (64/139, 46.0%) that were diagnosed correctly using Mode A and Mode B, respectively. The diagnostic correct rate of Mode B was higher than that of Mode A (*p* = 0.036) (Table 3).

### 3.7. Correlation Analysis

**Figure 4:**
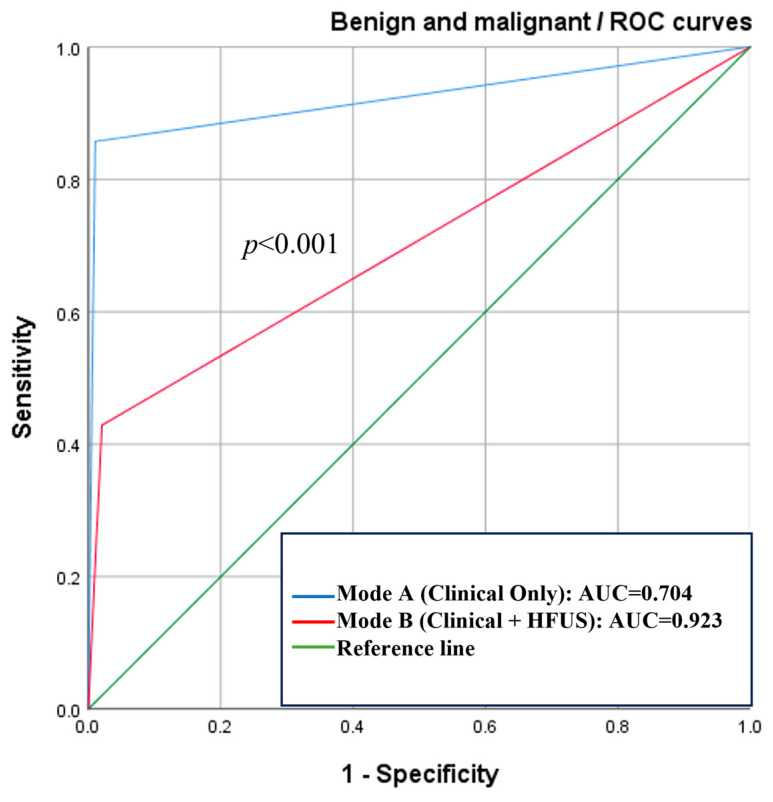
ROC curves of the benign and malignant lesions. The AUC value of Mode A is 0.704 (95% CI: 0.559~0.849) and of Mode B is 0.923 (95% CI: 0.834~1.000) (*n* = 119). It can be seen that Mode B exhibits superior performance.

Both modes identified 119 lesions out of 386 (119/386, 30.8%). For distinguishing between benign and malignant lesions, the ROC curves of the two modes are displayed in Figure 4. Though the specificity of Mode A is similar to that of Mode B (98.0% vs. 99.0%), the sensitivity of Mode B is significantly higher than that of Mode A (85.7% vs. 42.9%). Additionally, the AUC value of Mode B was significantly higher than that of Mode A [0.923 (95% CI: 0.834~1.000) vs. 0.704 (95% CI: 0.559~0.849), *p* < 0.001] (Table 4).

## 4. Discussion

The present study shows that HFUS can significantly enhance the diagnostic efficiency for unexplained soft tissue lesions at the end of the extremities, helping reduce unnecessary biopsies.

In our process of multi-classification analysis of lesions, HFUS is particularly effective in diagnosing common conditions and benign lesions, outperforming simple clinical physical examinations. Firstly, HUFS exhibits excellent performance in some common lesions, such as digital mucous cyst, cutaneous squamous cell carcinoma, malignant melanoma, wart, thecal cyst, and angioneoplasm. The diagnostic accuracy of HUFS for common and benign lesions reached 60.9% and 51.0%, respectively. As clinical doctors pay more attention to the diagnosis of common lesions, these conclusions are increasingly relevant to clinical practice. Additionally, while it may be less effective in detecting malignant lesions than benign ones, it demonstrates strong sensitivity, which aids in screening for malignancy. Meanwhile, due to the invisibility and difficulty in clinically palpating subungual lesions, it is challenging for doctors to make accurate diagnoses. For these lesions, diagnosis relies more on the doctor’s experience. Fortunately, our research has shown good results in diagnosing these lesions.

In our study, based only on clinical appearance and physical examination, the diagnostic accuracy is relatively low (17.9%). Using HFUS to diagnose these lesions has improved the diagnostic correct rate (52.8%). In our study, we found that HFUS reduced the diagnosis uncertainty while improving diagnostic accuracy. Specifically, Mode B effectively reduces uncertainty, which aids in the subsequent management of lesions (62.9% to 15.2%). Previously, Wortsman et al. conducted a retrospective study involving 4468 soft tissue masses located on various body parts, comparing ultrasound diagnoses with clinical diagnoses [22]. Their results indicated that providing HFUS images to clinicians can enhance the accuracy of skin lesions, aligning with the findings of our study. However, their study included only a limited type of lesions at the end of extremities, and only benign lesions were considered, which does not accurately reflect the real-world scenario. Therefore, a larger-scale study on lesions at the end extremities is necessary to validate the clinical feasibility of HFUS.

Bin Long et al. proposed a deep learning model for identifying superficial soft tissue masses throughout the body and achieved good diagnostic results [23]. In our study, the benign lesion diagnosis accuracy improved after incorporating HFUS information, marking a statistically significant improvement, especially in diagnosing cystic lesions, which are more sensitive, with a sensitivity rate of over 80% in our study. Also, the accuracy of angioneoplasm diagnosis has significantly improved with CDFI, reaching over 80.0%. This is similar to the results of Bin Long et al.’s study. However, due to the small number and wide variety of malignant cases, Bin Long et al.’s study only included five types of benign lesions. Although their mode has specific clinical application value, it does not include other types of lesions and limits the application prospects. Epidermal alterations can provide clinical doctors with more clinical information and are also the reason why most patients come to the hospital for treatment. However, due to the inherent limitations of our retrospective study, our dataset involved very few lesions without epidermal changes. We will prospectively collect epidermal data in ongoing studies to validate interactions between epidermal status and HFUS efficacy in order to improve our research in the future.

In our study, Mode B improved the diagnostic accuracy for radiologists with malignant lesions, but the difference between the two modes was not statistically significant. Interestingly, when analyzing specific disease types, squamous cell carcinoma and malignant melanoma were more accurately diagnosed among malignant lesions using Mode B (both *p* < 0.05). In addition, our study showed a higher diagnostic accuracy for Bowen’s disease in Mode B, which may be due to the typical HFUS imaging features of Bowen’s disease (limited to the epidermis, the “wave sign” of superficial high echogenicity in tumors, with a flat and clear boundary at the bottom of the cancer); thus, observers were generally able to diagnose Bowen’s disease accurately [24]. However, because the incidence rate of Bowen’s disease at the end of the extremities is low and the clinical manifestations are not typical, the clinical diagnosis is difficult [25]. Although the *p*-value between the two modes cannot be calculated in our study (possibly due to the limited number included), it is still stated that Mode B had excellent diagnostic accuracy for Bowen’s disease.

There was a different spectrum of diseases in different anatomical parts, and previous studies on finger and toe lesions mostly focused on case reports [26,27,28,29,30,31,32]. We analyzed the diagnostic performance of the two modes for finger and toe lesions, as well as for subungual and non-subungual lesions. Most subungual lesions are not visible in clinical practice; it is difficult for clinical doctors to diagnose them based solely on physical examination. Our results indicate that because of the HFUS penetration ability and visualization of internal information, Mode B showed good diagnostic results on nail lesions.

It should be pointed out that not all diseases can be examined by HFUS. In our study, there were 51 cases of lesions that were not visible on HFUS, mainly including melanocytic nevi of the nail matrix (*n* = 10), subungual melanoma in situ (*n* = 9), and junctional nevi (*n* = 8). Because of the relatively thin lesions (<0.1 mm), providing HFUS examination with the advantage of longitudinal information may lead to insufficient lesion assessment and failure to achieve ideal results. So, we believe that these lesions may require evaluation through other imaging or pathological examinations. Excluding cases undetectable by HFUS, Mode B diagnostic precision improved. Disparities persist between Mode A and Mode B. We believe that the limited number of undetectable cases does not undermine the overall effectiveness of HFUS in disease diagnosis. Among the 119 patients simultaneously diagnosed by physicians using both modes, Mode B exhibited superior clinical utility in differentiating malignant invasive lesions. HFUS can be used as a screening tool for malignant lesions, leveraging its advantages of a wide range, low cost, and high resolution [33].

One of the main limitations of our study is that it is a single-center retrospective study with a relatively small sample size, especially for malignant and rare lesions. Although the frequency of malignant and rare lesions in clinical practice is relatively low, their cumulative total may be considerable due to the variety of skin diseases. We refer to this phenomenon as the “long tail effect” [34]. Their clinical management is still crucial and cannot be ignored. Secondly, our study used pathology as the gold standard, but some patients who only require follow-up may not undergo biopsy or surgery, which may affect the proportion of each disease type. This has resulted in a relatively limited number of individual cases we have collected, affecting the statistical efficiency of some diseases. Thirdly, differences in clinical experience among doctors may lead to different diagnostic outcomes, which may affect the study’s statistical results. Clinical diagnosis prioritizes morphology and history (e.g., stable pigmented macule → benign nevus), while HFUS emphasizes structural invasion. When HFUS showed subtle irregularities in truly benign lesions (e.g., traumatic neuromas), radiologists favored malignancy. On the one hand, ultrasound provides an effective diagnostic tool for clinical doctors, helping them better diagnose diseases. On the other hand, ultrasound may also provide redundant information for clinical doctors, leading to misdiagnosis. We will continue to collect data on these cases to alleviate these limitations.

## 5. Conclusions

In summary, in the diagnostic evaluation of soft tissue lesions at the end of the extremities, the integration of high-frequency ultrasound (HFUS) with clinical data significantly bolsters diagnostic accuracy and mitigates diagnostic ambiguity. This synergistic approach is especially efficacious for common and benign lesions. Although its performance in malignant lesion diagnosis is suboptimal, HFUS demonstrates high sensitivity in detecting malignancies, rendering it valuable for screening purposes and minimizing the risk of missed diagnoses. However, its clinical utility is relatively limited for rare lesions. Overall, HFUS, when combined with clinical information, offers a comprehensive diagnostic paradigm for extremity soft tissue lesions.

## Figures and Tables

**Table 1:** Demographics and clinical characteristics (*n* = 386).

Characteristics	
No. of patients	386
Sex, *n* (%)	
Male	154 (39.9%)
Female	232 (60.1%)
Age (years)	50.4 ± 20.1

Data are presented as numbers (percentages %) and mean ± standard deviations.

**Table 2:** Type of lesions.

Type	Total (*n*)	Mode A			Mode B			*p* *
		T	F	Ind	T	F	Ind	
Digital mucous cyst	55	16 (29.1)	8 (14.5)	31(56.4)	45 (81.8)	9 (16.4)	1 (1.8)	<0.001
Angioneoplasm	52	23 (44.2)	7 (13.5)	22 (42.3)	45 (86.5)	5 (9.6)	2 (3.9)	<0.001
Malignant melanoma	32	9 (28.1)	8 (25.0)	15 (46.9)	19 (59.4)	3 (9.4)	10 (31.2)	0.003
Wart	22	6 (27.3)	4 (18.2)	12 (54.5)	12 (54.6)	5 (22.7)	5 (22.7)	0.039
Cutaneous squamous cell carcinoma	20	3 (15.0)	5 (25.0)	12 (60.0)	13 (65.0)	3 (15.0)	4 (20.0)	<0.001
Nevus	20	7 (35.0)	2 (10.0)	11 (55.0)	9 (45.0)	2 (10.0)	9 (45.0)	0.109
Nail matrix nevi	18	1 (5.6)	1 (5.6)	16 (88.8)	7 (38.9)	2 (11.1)	9 (50.0)	0.765
Thecal cyst	16	1 (6.3)	1 (6.3)	14 (87.4)	14 (87.4)	2 (12.6)	0 (0.0)	0.008
Papillary endothelial hyperplasia	14	0 (0.0)	3 (21.4)	11 (78.6)	5 (35.7)	8 (57.1)	1 (7.2)	/
Dermatofibroma	14	0 (0.0)	0 (0.0)	14 (100.0)	4 (28.6)	10 (71.4)	0 (0.0)	/
Subungual exostosis	12	0 (0.0)	3 (25.0)	9 (75.0)	8 (66.7)	4 (33.3)	0 (0.0)	0.236
Giant cell tumor of the tendon sheath	10	1 (10.0)	1 (20.0)	8 (80.0)	5 (50.0)	5 (50.0)	0 (0.0)	0.444
Granuloma	9	0 (0.0)	0 (0.0)	9 (100.0)	4 (44.4)	2 (22.2)	3 (33.4)	/
Bowen’s disease	8	0 (0.0)	3 (37.5)	5 (62.5)	7 (87.5)	1 (12.5)	0 (0.0)	/
Acquired fibrokeratoma of the finger/toe	5	1 (20.0)	2 (40.0)	2 (40.0)	1 (20.0)	3 (60.0)	1 (20.0)	0.600
Acral fibromyoma	8	0 (0.0)	4 (50.0)	4 (50.0)	0 (0.0)	3 (37.5)	5 (62.5)	/
Nail black spot	5	0 (0.0)	2 (40.0)	3 (60.0)	0 (0.0)	1 (20.0)	4 (80.0)	/
Chronic proliferative changes	5	0 (0.0)	3 (60.0)	2 (40.0)	0 (0.0)	1 (20.0)	4 (80.0)	/
Rare diseases ^	61	3 (4.9)	16 (26.2)	42 (68.9)	6 (9.8)	36 (59.0)	19 (31.1)	0.060
Total	386	71 (18.4)	72 (18.7)	243 (62.9)	204 (52.8)	105 (27.2)	77 (20.0)	<0.001

Data are presented as numbers (%) unless indicated otherwise. (T: correct diagnosis; F: misdiagnosis; Ind: indeterminate diagnosis). * Indicates a statistically significant difference between the two groups (*p* < 0.05). *p*-value that cannot be calculated is represented by “/”. ^ The rare diseases mainly include neurilemoma, fungal infection, periungual fibroma, eccrine poroma, calcium deposition disease, neurofibromatosis, etc.

**Table 3:** The classification of lesions.

Type	Total (*n*)	Mode A			Mode B			*p*
		T	F	Ind	T	F	Ind	
Total	386	71 (18.4)	72 (18.7)	243 (62.9)	204 (52.8)	105 (27.2)	77 (20.0)	<0.001 *
Incidence rate ^#^								
Common lesions	325	68 (20.9)	56 (17.2)	201 (61.9)	198 (60.9)	69 (21.2)	58 (17.9)	<0.001 *
Rare lesions	61	3 (4.9)	16 (26.2)	42 (68.9)	6 (9.8)	36 (59.0)	19 (31.2)	0.06
Benign and malignant lesions								
Benign	324	59 (18.2)	55 (17.0)	210 (64.8)	165 (51.0)	98 (30.2)	61 (18.8)	<0.001 *
Malignant	62	12 (19.4)	16 (28.8)	34 (54.8)	39 (62.9)	7 (11.3)	16 (25.8)	0.198
Location								
Subungual lesions	71	15(21.1)	11 (15.5)	45 (63.4)	29 (40.8)	11 (15.5)	31 (43.7)	0.009 *
Non-subungual lesions	315	56 (17.8)	61 (19.4)	198 (62.8)	175 (55.6)	94 (29.8)	46 (14.6)	<0.001 *
Anatomical								
Finger	247	55 (22.3)	50 (20.2)	142 (57.5)	140 (56.7)	68 (27.5)	39 (15.8)	<0.001 *
Toe	139	16 (11.5)	22 (15.8)	101 (72.7)	64 (46.0)	37 (26.7)	38 (27.3)	0.036 *
Visible lesions (Mode B)	335	65 (19.4)	58 (17.3%)	212 (63.3%)	204 (60.9)	105 (31.3%)	26 (7.8%)	<0.001 *

Data are presented as numbers (%) unless indicated otherwise. (T: correct diagnosis; F: misdiagnosis; Ind: indeterminate diagnosis). ^#^ Lesions are classified as common (*n* ≥ 5) or rare (*n* < 5) based on the incidence rate. * Indicates a statistically significant difference between the two groups (*p* < 0.05).

**Table 4:** Correlation analysis.

Type	Mode	Sensitivity	Specificity	AUC (95% CI)	Delong Test (*p*)
Malignant and benign lesions	A	42.9%	98.0%	0.704 (0.559~0.849)	<0.001 *
	B	85.7%	99.0%	0.923 (0.834~1.000)	

Data in brackets are the 95% confidence interval. Abbreviations: AUC, area under the receiver operating characteristic curve (*n* = 119). * Indicates a statistically significant difference between the two groups (*p* < 0.05).

**Figure A1:**
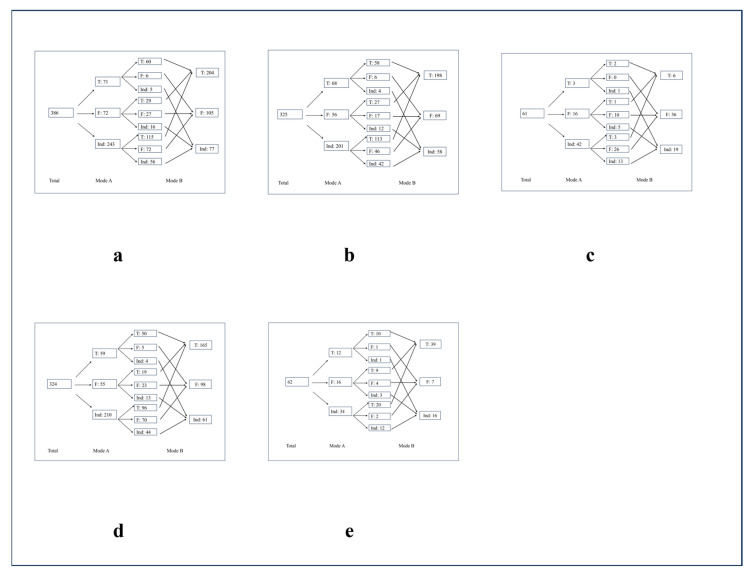
The attached diagram clearly illustrates the specific flow of the Sankey diagram. It is a supplementary explanation to Figure 2. The third column represents the direction of lesion diagnosis. The fourth column represents the final diagnostic result of Mode B. The (**a**–**e**) in the Figure A1 correspond to the (**a**–**e**) images in the Sankey diagram, which are used to explain the distribution of the Sankey diagram. ((**a**): All lesions; (**b**): Common lesions; (**c**): Rare lesions; (**d**): Benign lesions; (**e**): Malignant lesions).

## Data Availability

The datasets generated and/or analyzed during the current study are available from the corresponding authors upon reasonable request.

## Abbreviations

HFUS	High-frequency ultrasound
ROC	Receiver operating characteristic curve
AUC	Areas under the curve
CDFI	Color Doppler flow imaging

## Appendix A

**Table A1:** The Rare diseases.

Type	Total (*n*)	Mode A			Mode B		
		T	F	Ind	T	F	Ind
Neurilemoma	4	0 (0.0)	1 (25.0)	3 (75.0)	0 (0.0)	3 (75.0)	1 (25.0)
Fungal infection	4	0 (0.0)	1 (25.0)	3 (75.0)	0 (0.0)	2 (50.0)	2 (50.0)
Periungual fibroma	4	0 (0.0)	2 (50.0)	2 (50.0)	1 (25.0)	0 (0.0)	3 (75.0)
Eccrine poroma	2	1 (50.0)	1 (50.0)	0 (0.0)	1 (50.0)	1 (50.0)	0 (0.0)
Calcium deposition disease	2	0 (0.0)	0 (0.0)	2 (100.0)	2 (100.0)	0 (0.0)	0 (0.0)
Onychopapilloma	2	0 (0.0)	1 (50.0)	1 (50.0)	0 (0.0)	2 (100.0)	0 (0.0)
Neurofibromatosis (NF)	2	0 (0.0)	0 (0.0)	2 (100.0)	1 (50.0)	1 (50.0)	0 (0.0)
Lipoma	2	1 (50.0)	0 (0.0)	1 (50.0)	1 (50.0)	1 (50.0)	0 (0.0)
Myofibroma	2	0 (0.0)	1 (50.0)	1 (50.0)	0 (0.0)	1 (50.0)	1 (50.0)
Seborrheickeratosis	2	0 (0.0)	1 (50.0)	1 (50.0)	0 (0.0)	2 (100.0)	0 (0.0)
Morphea	2	0 (0.0)	0 (0.0)	2 (100.0)	0 (0.0)	0 (0.0)	2 (100.0)
Infection	2	1 (50.0)	0 (0.0)	1 (50.0)	0 (0.0)	0 (0.0)	2 (100.0)
Scar	1	0 (0.0)	0 (0.0)	1 (100.0)	0 (0.0)	1 (100.0)	0 (0.0)
HPV infection	1	0 (0.0)	0 (0.0)	1(100.0)	0 (0.0)	1(100.0)	0 (0.0)
Ankyloblastoma	1	0 (0.0)	0 (0.0)	1(100.0)	0 (0.0)	0 (0.0)	1(100.0)
Traumatic neuroma	1	0 (0.0)	0 (0.0)	1(100.0)	0 (0.0)	1(100.0)	0 (0.0)
Malignant proliferating trichilemmal tumor	1	0 (0.0)	0 (0.0)	1(100.0)	0 (0.0)	0 (0.0)	1(100.0)
Malignant eccrine poroma	1	0 (0.0)	0 (0.0)	1(100.0)	0 (0.0)	0 (0.0)	1(100.0)
Sarcoidosis	1	0 (0.0)	0 (0.0)	1(100.0)	0 (0.0)	1(100.0)	0 (0.0)
Osteochonfroma	1	0 (0.0)	1(100.0)	0 (0.0)	0 (0.0)	1(100.0)	0 (0.0)
Angiokeratoma	1	0 (0.0)	0 (0.0)	1(100.0)	0 (0.0)	1(100.0)	0 (0.0)
Angiolipoma	1	0 (0.0)	0 (0.0)	1(100.0)	0 (0.0)	0 (0.0)	1(100.0)
Benign lichenoid keratosis	1	0 (0.0)	0 (0.0)	1(100.0)	0 (0.0)	1(100.0)	0 (0.0)
Chronic fibroid and myofibromatous hyperplasia	1	0 (0.0)	0 (0.0)	1(100.0)	0 (0.0)	1(100.0)	0 (0.0)
Chronic inflammatory changes	1	0 (0.0)	1(100.0)	0 (0.0)	0 (0.0)	1(100.0)	0 (0.0)
Chronic hyperplastic inflammation	1	0 (0.0)	1(100.0)	0 (0.0)	0 (0.0)	1(100.0)	0 (0.0)
Corpus callosum	1	0 (0.0)	1(100.0)	0 (0.0)	0 (0.0)	1(100.0)	0 (0.0)
Smooth muscle hamartomas	1	0 (0.0)	0 (0.0)	1(100.0)	0 (0.0)	1(100.0)	0 (0.0)
Soft tissue chondroma	1	0 (0.0)	0 (0.0)	1(100.0)	0 (0.0)	1(100.0)	0 (0.0)
melanonychia	1	0 (0.0)	0 (0.0)	1(100.0)	0 (0.0)	1(100.0)	0 (0.0)
Lichenoid dermatitis	1	0 (0.0)	0 (0.0)	1(100.0)	0 (0.0)	1(100.0)	0 (0.0)
Pemphigus	1	0 (0.0)	0 (0.0)	1(100.0)	0 (0.0)	1(100.0)	0 (0.0)
Gout	1	0 (0.0)	1(100.0)	0 (0.0)	0 (0.0)	1(100.0)	0 (0.0)
Angiokeratoma	1	0 (0.0)	0 (0.0)	1(100.0)	0 (0.0)	1(100.0)	0 (0.0)
Intravascular papillary endothelial hyperplasia	1	0 (0.0)	0 (0.0)	1(100.0)	0 (0.0)	1(100.0)	0 (0.0)
Angiopericytoma	1	0 (0.0)	0 (0.0)	1(100.0)	0 (0.0)	1(100.0)	0 (0.0)
Acroangiodermatitis	1	0 (0.0)	0 (0.0)	1(100.0)	0 (0.0)	1(100.0)	0 (0.0)
Atypical fibrous histiocytoma	1	0 (0.0)	0 (0.0)	1(100.0)	0 (0.0)	1(100.0)	0 (0.0)
Molluscumfibrosum	1	0 (0.0)	0 (0.0)	1(100.0)	0 (0.0)	0 (0.0)	1(100.0)
Acrokeratoelastoidosis	1	0 (0.0)	1(100.0)	0 (0.0)	0 (0.0)	1(100.0)	0 (0.0)
Reactive angioendotheliomatosis	1	0 (0.0)	1(100.0)	0 (0.0)	0 (0.0)	0 (0.0)	1(100.0)
Unilateral lentiginosis	1	0 (0.0)	0 (0.0)	1(100.0)	0 (0.0)	0 (0.0)	1(100.0)
Myopericytoma	1	0 (0.0)	0 (0.0)	1(100.0)	0 (0.0)	0 (0.0)	1(100.0)
Rare diseases	61	3 (4.9)	16 (26.2)	42 (68.9)	6 (9.8)	36 (59.0)	19 (31.1)

Data are presented as numbers (%) unless indicated otherwise. (T: correct diagnosis; F: misdiagnosis; Ind: indeterminate diagnosis).
